# Multiple Assembly Rules Drive the Co-occurrence of Orthopteran and Plant Species in Grasslands: Combining Network, Functional and Phylogenetic Approaches

**DOI:** 10.3389/fpls.2016.01224

**Published:** 2016-08-17

**Authors:** Bertrand Fournier, Arnaud Mouly, François Gillet

**Affiliations:** ^1^Laboratoire Chrono-Environnement UMR 6249 CNRS, Université Bourgogne Franche-Comté, BesançonFrance; ^2^Jardin botanique de la ville de Besançon et de l’Université de Franche-Comté, BesançonFrance; ^3^Ecological Systems Laboratory, Ecole Polytechnique Fédérale de Lausanne, LausanneSwitzerland

**Keywords:** coexistence, competition, environmental filtering, functional traits, grasshoppers, grassland-invertebrate interactions, null models

## Abstract

Understanding the factors underlying the co-occurrence of multiple species remains a challenge in ecology. Biotic interactions, environmental filtering and neutral processes are among the main mechanisms evoked to explain species co-occurrence. However, they are most often studied separately or even considered as mutually exclusive. This likely hampers a more global understanding of species assembly. Here, we investigate the general hypothesis that the structure of co-occurrence networks results from multiple assembly rules and its potential implications for grassland ecosystems. We surveyed orthopteran and plant communities in 48 permanent grasslands of the French Jura Mountains and gathered functional and phylogenetic data for all species. We constructed a network of plant and orthopteran species co-occurrences and verified whether its structure was modular or nested. We investigated the role of all species in the structure of the network (modularity and nestedness). We also investigated the assembly rules driving the structure of the plant-orthopteran co-occurrence network by using null models on species functional traits, phylogenetic relatedness and environmental conditions. We finally compared our results to abundance-based approaches. We found that the plant-orthopteran co-occurrence network had a modular organization. Community assembly rules differed among modules for plants while interactions with plants best explained the distribution of orthopterans into modules. Few species had a disproportionately high positive contribution to this modular organization and are likely to have a key importance to modulate future changes. The impact of agricultural practices was restricted to some modules (3 out of 5) suggesting that shifts in agricultural practices might not impact the entire plant-orthopteran co-occurrence network. These findings support our hypothesis that multiple assembly rules drive the modular structure of the plant-orthopteran network. This modular structure is likely to play a key role in the response of grassland ecosystems to future changes by limiting the impact of changes in agricultural practices such as intensification to some modules leaving species from other modules poorly impacted. The next step is to understand the importance of this modular structure for the long-term maintenance of grassland ecosystem structure and functions as well as to develop tools to integrate network structure into models to improve their capacity to predict future changes.

## Introduction

Understanding the rules underlying species assembly is a key challenge in ecology (HilleRisLambers et al., 2012). In a foodweb, species interact hierarchically with species from other trophic levels through trophic interactions (producer-consumer, predator-prey, and parasite-host). Within trophic levels, different theories such as the competition, the environmental filtering and the neutral theories describe species assembly into ecological communities (*sensu* Hubbell, 2001). Practically, assembly rules within and among trophic levels were mainly considered separately. Studies have investigated the importance of among-guild interactions including plant-pollinator (Olesen et al., 2007), trophic networks (Dunne et al., 2002a) or host-parasite networks (Vázquez et al., 2005) without considering within-guild processes. Similarly, studies focusing on within-guild assembly rules have mainly focused on answering which of competition, environmental filtering and neutral processes could explain observed ecological assemblages (Cottenie, 2005). However, a growing body of evidence suggests that different species assembly mechanisms can operate simultaneously and that they should be placed along a continuum (Amarasekare et al., 2004; Mouquet et al., 2005; Gravel et al., 2006; Leibold and McPeek, 2006; Fournier et al., 2016). Understanding this complexity of processes is key to better predict and manage species assemblages and their associated functions and services (Balvanera et al., 2006; Cadotte et al., 2011) and becomes increasingly critical in the current context of global change and biodiversity crisis (Koh et al., 2004). However, it remains difficult to assess multiple assembly rules from species distribution or co-occurrence data (Fournier et al., 2016). Here, we explore to what extent the combination of species co-occurrence network and functional and phylogenetic approaches can provide new insights on how multiple rules interact to shape species assembly.

Phylogenetic relatedness and functional traits are strong determinants of the structure of ecological networks (Cattin et al., 2004; Martín González et al., 2015) that can be used to identify assembly rules (Götzenberger et al., 2012). According to the environmental filtering hypothesis, species lacking specific adaptations to local conditions are filtered from the community (Weiher et al., 1998; Cornwell et al., 2006). As a result, species with similar functional traits co-occur preferentially. If these traits are more similar among closely related species (phylogenetically conserved), closely related species should co-occur more often than expected by chance (Webb et al., 2002). Under the competition theory, the best local competitors are expected to exclude other species resulting in spatial or temporal partitioning of species distribution (Chesson, 2000; Grime, 2006). Eventually, this process can induce a selective pressure forcing the displacement of functional traits where sufficiently different species can coexist (limiting similarity) (MacArthur and Wilson, 1967; Wilson, 2007; Wilson and Stubbs, 2012). When functional traits are phylogenetically conserved, communities are expected to be composed by functionally dissimilar and phylogenetically unrelated species. Neutral drift of species abundance can also occur and support coexistence over extended periods (Hubbell, 2001). In this case, the functional and phylogenetic similarity among species is expected to be random.

In this paper, we explore the possibility of combining functional and phylogenetic analyses of assembly rules with ecological network approaches to go beyond the view of a single mechanism driving a whole assemblage. Our hypothesis is that co-occurrence networks constitute a directly observable outcome of species assembly whose structure results from different assembly rules. We focus here on two well-documented network structure: nestedness and modularity (Fortuna et al., 2010). Nestedness and modularity plays a key role for the stability of species-rich ecosystems and their response to global change (Bascompte and Stouffer, 2009). Modularity refers to the organization of a network into modules or groups where species co-occur more frequently within than among modules (Newman, 2006). Modularity can retain the impact of perturbations or land use changes within few modules thereby minimizing the impact on other modules (Krause et al., 2003; Teng and McCann, 2004). Well-known examples include pollination network in tropical high-altitude grasslands (Danieli-Silva et al., 2012) and the hummingbird–plant networks across the Americas (Martín González et al., 2015). Nestedness describes the organization of a network where species-poor assemblages are a subset of species-rich assemblages. It can make the community more robust to both random extinctions (Memmott et al., 2004; Burgos et al., 2007) and habitat loss (Fortuna and Bascompte, 2006). Nestedness was first described for insular fauna where island size strongly determines the total diversity. Examples for grasslands include temporal nestedness in Californian plant communities (Elmendorf and Harrison, 2009) or spatial nestedness in European butterfly communities (Öckinger and Smith, 2006). Assembly rules can change with the structure of the network (Bascompte et al., 2003; Bascompte and Stouffer, 2009). They can differ among modules in the case of a modular network or from the richer to the poorer assembly in the case of a nested network.

Here, we focus on the co-occurrence network of plant and orthopteran species in the grasslands of the French Jura Mountains. Semi-natural grasslands are biodiversity hotspots that provide important services to human societies such as food production or soil protection. These ecosystems typically host a large number of species over short spatial scales. Before the Middle Ages, the Jura Mountains were mostly covered by forests. Silvopastoral practices have reorganized species co-occurrence networks leading to the creation of grassland and wood-pasture ecosystems (Buttler et al., 2009). Nowadays, human activities increasingly threaten grassland biodiversity and thereby the organization of ecological networks. Plants and orthopterans are key actors of grasslands ecosystems. Orthopteran communities constitute an important link within grassland food chain. They are important consumers of plant biomass (Deraison et al., 2015) and their richness and abundance can impact higher trophic levels such as birds (Hamer et al., 2006). As such they can mediate trophic cascades and their consequences on element cycling (Strickland et al., 2013). Plants provide resources and habitats to a broad range of species and they fulfill key functions (production of biomass) that sustain important services to human societies (cattle foraging). Studying the co-occurrences of plants and herbivore insects thus provides important information about the functioning of grassland ecosystems. Furthermore, understanding how plant and orthopteran species assemble can provide important insight about how future changes will influence grassland ecosystems.

We first verified whether the plant-orthopteran co-occurrence network has a nested and/or modular structure. As our study encompasses an altitudinal gradient and clear changes in agricultural practices (Mauchamp et al., 2014), we expect the plant-orthopteran co-occurrence network to have a modular structure that reflects this environmental heterogeneity (Olesen et al., 2007). We then verified our main hypotheses that the modular structure of the co-occurrence network reflects a complexity of assembly processes. More specifically, species coexistence in some modules is expected to result from a filtering effect of intensive agricultural practices while species coexistence in other modules is expected to result from biotic interactions such as competition for resources among plants or orthopterans. To do so, we assessed the changes in functional traits and assembly rules (null models of species functional and phylogenetic similarity) among modules. We also expected agricultural practices, soil conditions and spatial variables to have a strong importance for the modular structure of the network. We used variance partitioning to assess the importance of these variables for the whole network as well as for each module individually.

**Table 1:** Selected traits of **(A)** orthopteran and **(B)** plant species.

Trait	Short name	Values	Definition
**(A)**			
Habitat specificity	Habitat	0 = narrow, 1 = wide	Range of habitats of a species
Dispersal capacity	Dispersal	0 = limited, 1 = high	Capacity to disperse
Change in feeding regime	Feed_change	0 = no; 1 = yes	Change in feeding regime during life cycle
Egg deposition preference	Egg_deposition	0 = soil, 1 = plants	Preferred location for egg deposition
**(B)**			
Maximum height	Hmax	[cm]	Maximum height of a plant species
Leaf dry matter content	LDMC	%	% of leaf biomass remaining after desiccation
Seed mass	Seed_mass	[mg]	Seed mass in mg
Specific leaf area	SLA	0–1	Ratio of leaf area to dry mass

## Materials and Methods

### Study Site and Sampling Design

**FIGURE 1:**
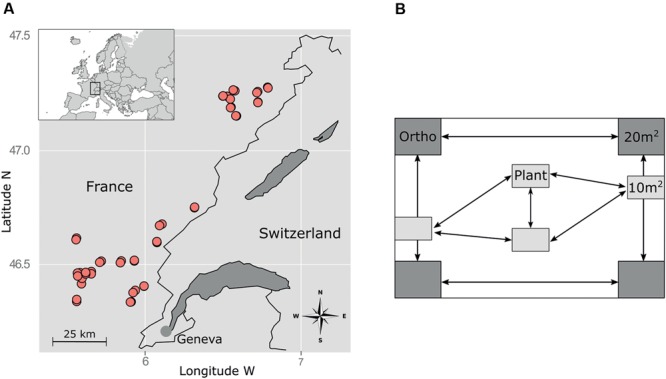
**Sampling design. (A)** Location of the study area (upper left corner) and spatial distribution of the plots within the study area. **(B)** Sampling design within each plot. Plant and orthopteran communities were sampled in the same year (2011) and in the same plots but at different periods (following the shift in optimal development of vegetation and insect populations) and at different subplot locations to avoid disturbances by observers.

The study was conducted in the NW part of the French Jura Mountains in an area located between 391 and 1195 m a.s.l. and characterized by a nemoral climate with a strong suboceanic influence (**Figure 1A**). The dominant soils are cambisols developed on limestone. Permanent grasslands cover about 22% of the total surface of the study area. They are mainly used for dairy farming and Protected Designation of Origin cheese production (mainly Comté cheese, a major economic sector, with constraining specifications for agricultural practices). Within this area, we targeted mesic grasslands that have not been plowed and sown at least for the 10 past years and where it was possible to delimit a 1000 m ×1000 m rectangular plot located on a flat area. These selection criteria allowed us to avoid potential biases due to slope or extreme soil conditions (excluding wet or dry grasslands) so as to focus on the effect of agricultural practices (grazing, mowing, fertilization) and climatic conditions (elevation) on plant and orthopteran communities. Overall, 24 farmers accepted to participate by indicating two parcels per farm, one mainly used as pasture and one as hayfield, resulting in a total of 48 grasslands that met our criteria. The 48 grasslands encompassed a gradient of mowing and grazing practices (from strictly grazed to frequently mown including variations in grazing intensity and mowing frequency) as well as various fertilization regime (from no fertilizers to high input of fertilizers; fertilizer type: liquid or solid manure, organic or industrial fertilizers) (Mauchamp et al., 2014).

### Agricultural Practices and Soil Surveys

Agricultural practices were assessed for each plot by interviewing the farmers. We used a questionnaire aiming at defining the defoliation regime and nutrient input regime. Questions concerned grassland management (mowing frequency, forage yield, grazing duration, livestock type, stocking rate) as well as the amount and type of fertilizers (liquid or solid manure, industrial fertilizers) applied during the year preceding sampling as well as during the last 10 years. Defoliation regime was assessed by the mean number of cuts per year (*cutting*, 0 in strictly grazed parcels), and by the stocking rate (*grazing*) expressed in livestock units days per hectare and per year (available for year 2011 only). The fertilization regime was evaluated by the mean amounts of available nitrogen brought per hectare and per year, by all potential sources (liquid and solid manure and industrial fertilizers), averaged over the 10 past years (*fertilizers*).

Soil surveys were carried out in each plot to assess soil texture and chemical composition. A total of eight soil subsamples were taken in each plot to account for within-plot heterogeneity. These samples were then pooled for analyses of total N, C/N and soil cation exchange capacity (CEC).

### Insect and Plant Sampling

We sampled orthopterans in August–September 2011 in four 20-m^2^ subplots located in the four corners of each 1000-m^2^ plot (**Figure 1B**). In each subplot, we conducted 100 sweeps using a standard net of 40 cm in diameter. We then conducted 5 min of hand searching to target the remaining individuals. All adult individuals were frozen and identified to species level (Dehondt and Mora, 2013). Trait data were gathered in the literature (Hendriks et al., 2013; Gossner et al., 2015) (**Table 1A**).

The vegetation of the 48 selected grasslands was sampled in May–June 2011 in four rectangular subplots of 10 m^2^ (4 m × 2.5 m). These plots were placed on the flattest area inside the parcel, presenting a homogeneous vegetation physio gnomy and far from the parcel’s margin. All vascular plant species observed in each plot were listed and the relative cover of each species was estimated using the seven degrees of the Braun-Blanquet’s scale. Plant trait data were gathered in various databases (Jäger, 2000; Kühn et al., 2004; Kleyer et al., 2008; Klimešová and De Bello, 2009; Mauchamp et al., 2014) (**Table 1B**).

### Phylogenetic Data

For orthopteran taxa, we searched DNA sequences of the cytochrome oxidase subunit 1 (COI) on Genbank for each species observed. When no sequences were available for the target species, we used species from the same genus as surrogate. Species from the same genus were available in all cases and we thus did not need to go to family level. We used the sequences of all species of the same genus as the target species to calculate an average phylogenetic distance between the target genus and the other species. We used ClustalX 2.1 (Larkin et al., 2007) and Se-Al 1.0al software (Rambaut, 1996, University of Oxford, Oxford, UK^1^) to align the sequences. We analyzed these datasets using a combined Bayesian Monte Carlo Markov Chain approach under BEAST 1.5.3 (Drummond and Rambaut, 2007). We then performed AIC-based selection of the model of nucleotide substitution using MrModeltest 2.0 (v 2.0, Evolutionary Biology Centre, Uppsala University, Sweden). Several family relationships were constrained according to Song et al. (2015) in BEAST to calibrate the rates of molecular evolution of each lineage. We consequently assessed the regional phylogeny by building an ultrametric maximum likelihood tree using mantid sequences (*Apteromantis aptera, Tamolanica tamolana* and *Ameles* sp.) as outgroup. We use the obtained tree to calculate the cophenetic distances among all pairs of species. We obtained the plant phylogenetic distance matrix from an ultrametric multiple-genes regional tree for the 197 plant species recorded in the study area (Mauchamp et al., 2014). We searched Genbank for data about two genes encoding chloroplast proteins (rbcL and matK).

### Co-occurrence Network

**FIGURE 2:**
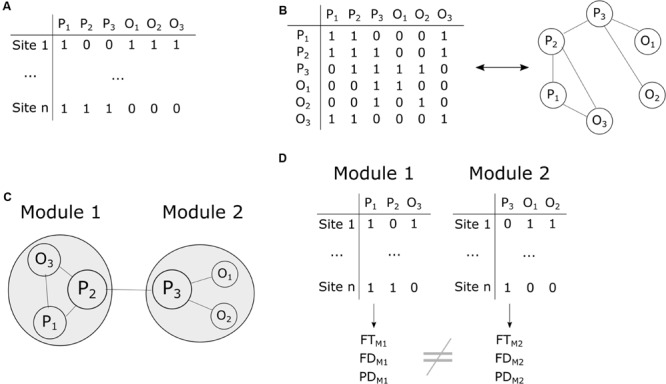
**Summary of the different steps of the co-occurrence data analyses. (A)** The input site × species matrix contains information about species presence–absence (1-0) in each plot. P_1_, P_2_, P_3_ denote plant species and O_1_, O_2_, O_3_ orthopteran species. **(B)** The presence-absence matrix is transformed into a co-occurrence species × species matrix where 1 means positive co-occurrence. In turn, the co-occurrence matrix is used to build a co-occurrence network. **(C)** Assessment of network modularity and nestedness (here a hypothetical modular network with two modules is shown) as well as species importance for network structure (circle size is proportional to species importance). **(D)** Assessment of functional traits (FT), functional distances (FD) and phylogenetic distances (PD) within modules and comparisons among modules.

We classified all pairs of species as having positive, negative or random co-occurrences using the probabilistic model of Veech (2013) (**Figures 2A,B**). This model calculates the observed and expected probabilities of co-occurrence of all pairs of species and determines whether the observed values is lower or higher than expected by chance. In the case of a positive co-occurrence, two species are more frequently encountered together than expected by chance. To the contrary, two species have a negative co-occurrence when they are more frequently encountered alone than expected by chance. We focused on positive co-occurrences to build an undirected network. The final co-occurrence matrix included all species in rows and columns and was filled with 0 in the absence of positive co-occurrence and 1 otherwise. Species showing no positive co-occurrence were filtered from the data at this stage.

We assessed the nestedness and modularity of the resulting network (**Figure 2C**). Nestedness was estimated using the weighted index of Galeano et al. (2009) where 1 represents perfect nestedness and 0 no nested structure. The classification of species into modules was obtained using the algorithm of Dormann and Strauss (2014). Modularity was then measured with the Newman’s Q index of modularity (Clauset et al., 2004; Newman, 2004) where values above 0.3 are good indicators of significant structuring of the network. We then assessed whether the observed nestedness and modularity values were lower or greater than expected by chance. To do so, we computed 9,999 permutations of the co-occurrence network and computed nestedness and modularity for each iteration. The resulting values provided a null distribution of nestedness and modularity values that was used to compute standard effect sizes and *p*-values. To minimize potential bias related to the chosen methodology, we compared the ‘swap’ (Gotelli and Entsminger, 2003), ‘tswap’ (Miklós and Podani, 2004) and ‘quasiswap’ (Miklós and Podani, 2004) permutation algorithms where row and columns sums are fixed in all cases.

### Numerical Analyses

We assessed the contribution of each species to the structure of the co-occurrence network using a knock-out approach (**Figure 2C**). We removed all species one by one from the data and calculated the modularity and nestedness of the network. We subtracted the obtained values (n-1 species) from the initial values of modularity and nestedness (*n* = 91 species) to obtain Δ_i_ for each species *i*. Δ_i_ was transformed into Standardized Effect Size (SES*_i_*) according to:

$${SES}_{i}=\left(\Delta_{i}-\frac{\Sigma_{j=1}^{n}\Delta_{j}}{n}\right)/\sigma$$

where σ is the standard deviation of Δ_j_.

We then used the plant and orthopteran functional and phylogenetic distance matrices to investigate whether assembly rules change with the structure of the network (**Figure 2D**). We tested individually for the different parts of the network (the different modules in the case of a modular network or the species-rich and species-poor assemblies in the case of a nested network) whether functional and phylogenetic distances among species were greater or lower than expected by chance. To do so, we randomly attributed species to the different parts of the network and calculated the mean functional and phylogenetic distances for each network part. This procedure has the advantage of preserving the structure of the distance matrices. We applied a similar procedure to functional distance matrices computed using all traits (multiple trait) as well as to functional distance matrices computed with each trait individually (individual trait). We also used Kruskal–Wallis non-parametric tests to assess whether the distribution of plant and orthopteran functional traits changes with the structure of the network.

We compared this co-occurrence-network approach to an abundance-based approach. The goal here was to assess whether the rules underlying species co-occurrences and abundance are similar. In this case, we used the dispersion index of Laliberté and Legendre (2010) using the functional and phylogenetic distance matrices as input. We thereby obtained an index of functional dispersion (FDis) and an index of phylogenetic dispersion (PDis) that were used for null-model testing. We computed 9,999 permutations of the species abundance matrix using the same procedure as described above and calculated simulated values of FDis and PDis. We used the resulting distribution to calculate SES and *p*-values. This procedure preserves local abundance and diversity, yet attributing random abundance to species.

We also assessed to what extent spatial, soil and agricultural variables explain species abundance data. We computed variance partitioning based on RDA (Borcard et al., 2011) for all species (i.e., ignoring network structure) as well as for each module separately. Species abundance data were Hellinger-transformed prior to analyses (Legendre and Gallagher, 2001). To conduct variance partitioning analyses for each module, we first divided the initial species abundance matrix into five subsets each containing information about the species of a single module only. We then conducted variance partitioning for each of these subsets. We finally compared the proportion of the variance in abundance data explained by the three sets of environmental variables in the whole network to that in the different parts of the network.

**FIGURE 3:**
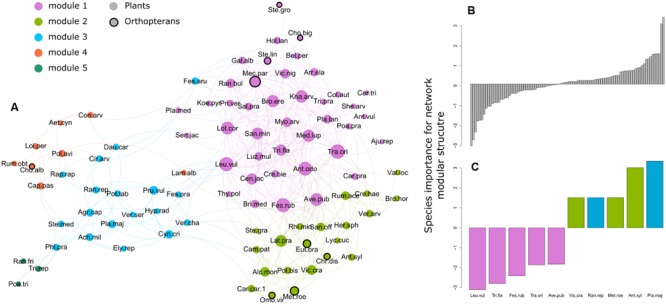
**(A)** Plant and orthopteran species co-occurrence network in the permanent mesic grasslands of the French Jura Mountains. Points (edges) correspond to species and lines (vertex) to significant positive co-occurrence between two species. Point size is proportional to species degree (i.e., the total number of significant positive co-occurrences with the species). Colors show species membership to the five modules revealed by the modularity analysis. Abbreviation corresponds to the three first letters of the genus and species names following Supplementary Table S1 (online Supplementary Material). **(B)** Species importance for the modular structure of the network. Values on the *y*-axis are standardized effect sizes. Negative values indicate species with negative impact on modularity (i.e., the network becomes more modular when the species is removed) and positive values indicate species with positive impact. **(C)** Zoom on the five species with the highest negative and positive impact on modularity, respectively. Colors show species module membership.

Network analyses were done with packages “igraph” (Csardi and Nepusz, 2006) and “co-occur” (Veech, 2013) of R-3.2.1 (R Development Core Team, 2015). Packages “FD” (Laliberté and Legendre, 2010) and “picante” (Kembel et al., 2010) were used for functional and phylogenetic analyses, respectively. Package “vegan” (Oksanen et al., 2015) was used for variance partitioning. Network visual representation (**Figure 3A**) was done in Gephi (Bastian et al., 2009).

## Results

We sampled 22 and 197 species of orthopterans and vascular plants, respectively. The dominant and more frequently encountered species in orthopteran communities were *Chorthippus parallelus* and *Chorthippus biguttulus*. *Poa trivialis*, *Trifolium repens* and *Taraxacum officinale* were the dominant plant species.

After trimming species lacking positive co-occurrences, 82 plant and 9 orthopteran species remained. These represented 42 and 41% of the total number of plant and orthopteran species, respectively. The resulting network had a relatively high modularity (0.36), but a low nestedness value (0.29) (**Figure 3A**). Our permutation analyses revealed a significantly higher modularity than expected by chance (SES = 18.8; *P* < 0.001) with five groups of co-occurring species. To the contrary, the observed nestedness was significantly lower than expected by chance (SES = -5.2; *P* = 0.01). The most connected plant species (i.e., species having numerous significant positive co-occurrences with other species) were *Tragopogon orientalis, Leucanthemum vulgare, Festuca rubra and Anthoxanthum odoratum* in module 1, *Lathyrus pratensis*, *Vicia cracca* and *Alchemilla monticola* in module 2, *Prunella vulgaris* and *Cynosurus cristatus* in module 3. Orthopterans were present in module 1 (*Chorthippus biguttulus, Mecostethus parapleurus, Stenobothrus lineatus* and *Stethophyma grossum*) and 2 (*Chrysochraon dispar, Euthystira brachyptera, Metrioptera roeselii* and *Omocestus viridulus*) and in module 4 with *Chorthippus albomarginatus*. Species with the strongest positive influence on modularity were found in modules 2 and 3 (e.g., *Metrioptera roeseli*) while those with the strongest negative influence on modularity belonged to module 1 (**Figures 3B,C**).

**FIGURE 4:**
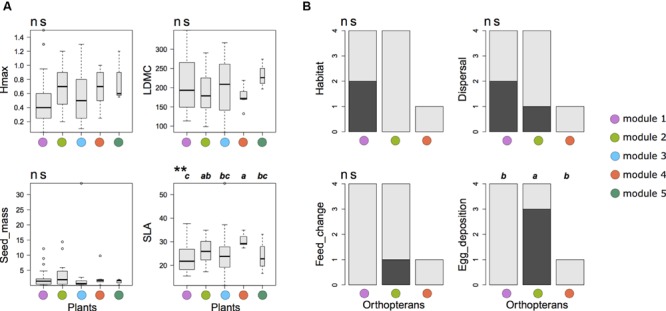
**Functional trait variations among the modules of the plant-orthopteran co-occurrence network of the French Jura Mountains. (A)** Variation in plant functional traits. Hmax, maximal vegetative height; LDMC, leaf dry matter content; Seed_mass, seed mass; SLA, specific leaf area. Kruskal–Wallis non-parametric tests were used to assess significant differences among modules (**P < 0.01; ns *P* > 0.05). **(B)** Variation in orthopteran functional traits. The *y*-axis shows the number of species with a given trait value (light gray = 0 and dark gray = 1). *Chorthippus albomarginatus* was the only species present in module 4. Habitat: range of occupied habitats (0 = narrow; 1 = wide); Dispersal: capacity to disperse (0 = limited, 1 = high); Feed_change: change of feeding habits during life cycle (0 = no, 1 = yes); Egg_deposition: preferred location for egg deposition (0 = soil, 1 = plants). Differences were tested using Fisher’s exact test. Only egg deposition showed a marginally significant difference among modules (*P* = 0.09).

Null-model tests on co-occurrence data revealed a significantly higher phylogenetic and functional distance among plants than expected by chance in modules 3 and 5, respectively (**Table 2A**). By contrast, the same analysis revealed a significantly lower functional distance than expected by chance in module 4 for multiple trait, SLA and LDMC (**Tables 2A,B**). Plant species in this module had a higher average SLA with a lower variance than any other modules (**Figure 4A**). Similarly, the three species in module 5 were phylogenetically less related than expected by chance. Null-model tests on abundance data without considering network structure revealed a lower functional distance among species than expected by chance for multiple traits, LDMC and seed mass (**Table 3A**). For orthopterans, null-model tests on co-occurrence data were limited to modules 1 and 2 where several species were present and revealed no significant pattern in functional or phylogenetic data (**Tables 2A,C**). The same was true for abundance data (**Table 3B**). This agrees with the lack of significant changes of functional traits among modules except for a marginally significant change in egg deposition strategy (**Figure 4B**). Nevertheless, orthopterans in module 1 tended to be generalists with broad environmental range and good dispersal capacity. In module 2, species were preferentially habitat specialists with intermediate dispersal capacity and changed their diet during life cycle and preferentially lay eggs in plants. This group was composed by species characteristic of mountain grasslands such as *Metrioptera roeseli*. Finally, *Chorthippus albomarginatus*, the only orthopteran in its module, is a habitat specialist with a low dispersal capacity.

**Table 2:** Null model analysis of plant and orthopteran functional and phylogenetic distances in the five species groups (Modules 1–5) revealed by the modularity analysis.

(A)		Functional distances				Phylogenetic distances			
	Plant		Orthopteran		Plant		Orthopteran	
	SES	*P*	SES	*P*	SES	*P*	SES	*P*
Module 1	-1.11	0.148	-0.26	0.363	0.33	0.60	-0.75	0.349
Module 2	-0.3	0.394	-0.35	0.375	**-2.20**	**0.03**	0.4	0.737
Module 3	**1.83**	**0.957**			0.07	0.49		
Module 4	**-1.77**	**0.028**			0.05	0.44		
Module 5	-0.02	0.553			**1.32**	**0.98**		

**(B)**	**Hmax**		**LDMC**		**Seed_mass**		**SLA**	
	**SES**	***P***	**SES**	***P***	**SES**	***P***	**SES**	***P***

Module 1	-0.32	0.366	1.02	0.848	-0.91	0.211	-1.46	0.074
Module 2	-0.46	0.351	-0.36	0.361	0.67	0.790	-0.93	0.147
Module 3	-0.38	0.332	1.09	0.853	0.73	0.759	1.88	0.948
Module 4	0.41	0.642	-**2.42**	**0.010**	-0.09	0.580	**-1.56**	**0.015**
Module 5	0.27	0.638	-0.60	0.306	-0.45	0.444	0.94	0.864

**(C)**	**Habitat**		**Dispersal**		**Feed_change**		**Egg_deposition**	
	**SES**	***P***	**SES**	***P***	**SES**	***P***	**SES**	***P***

Module 1	1.12	0.924	0.83	0.820	-0.92	0.271	-2.48	0.061
Module 2	-1.59	0.137	0.00	0.387	1.15	0.785	0.03	0.387

***(A)** Functional distances based on multiple traits. **(B)** Null-model analysis of plant functional distances based on individual traits. **(C)** Null-model analysis of orthopteran functional distance based on individual traits. SES, standardized effect size; *P* = *P*-values after 9,999 permutations of the distance matrices. Significant negative or positive SES are in boldface.*

**Table 3:** Abundance-based null model analysis of plant and orthopteran functional and phylogenetic distances for the whole dataset.

(A)			(B)		
	Plants			Orthopterans	
	SES	*P*		SES	*P*
Multi-traits	**-1.72**	**0.022**	Multi-traits	0.45	0.713
Hmax	-0.56	0.31	Habitat	1.62	0.89
LDMC	**-1.67**	**0.04**	Dispersal	0.56	0.729
Seed_mass	**-1.21**	**0.028**	Feed_change	0.26	0.783
SLA	-0.15	0.505	Egg_deposition	-1.25	0.128
Phylogenetic	-1.06	0.132	Phylogenetic	0.09	0.705

***(A)** Plants. **(B)** Orthopterans. SES, standardized effect size; *P* = *P*-values after 9,999 permutations of the distance matrices. Significant negative or positive SES are in boldface.*

**FIGURE 5:**
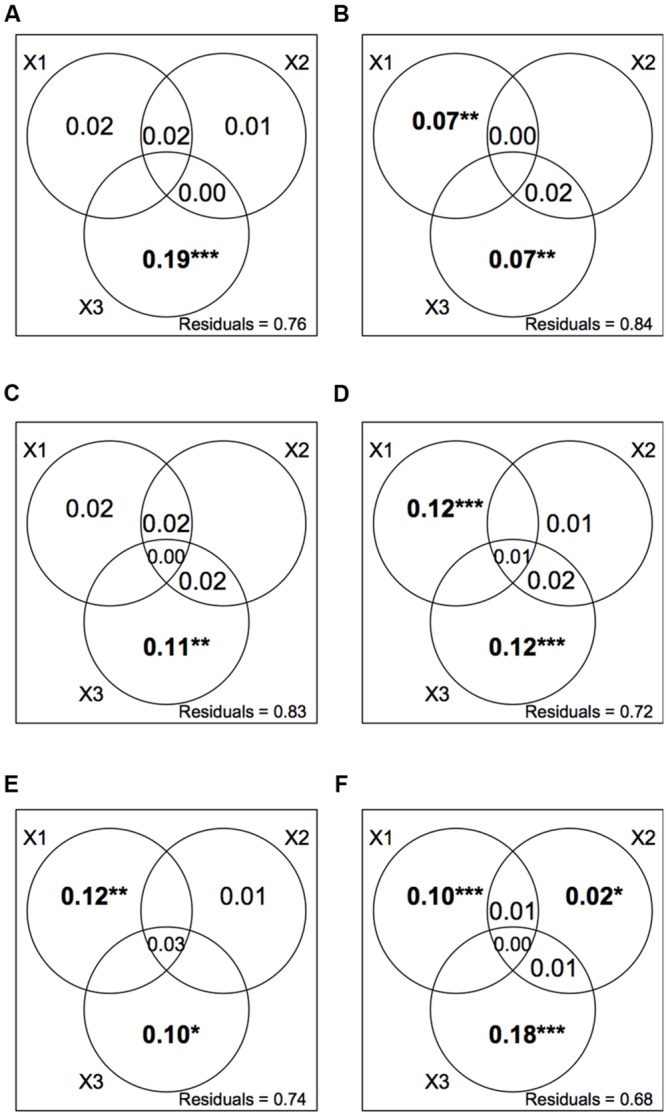
**Variance partitioning of plant and orthopteran abundance data explained by three sets of explanatory variables; X1: agricultural practices (*cutting*, the mean number of cuts per year; *grazing*, the stocking rate; *fertilizers*, the total amount of N input); X2: soil conditions (total N, C/N, CEC); X3: spatial location (longitude; latitude; elevation).** We show the adjusted R^2^ for all non-negative fractions. **(A–E)** Individual variance partitioning for modules 1– 5. **(F)** Variance partitioning for the whole network. Stars and bold typeface indicate significance (***P < 0.001; **P < 0.01; *P < 0.05).

Variance partitioning of plant and orthopteran abundance data revealed that spatial variables were good predictors of species distribution in all five modules as well as for the entire dataset (**Figure 5**). Agricultural practices were significant predictors of species abundance in modules 2, 4, and 5 as well as for the entire dataset. Soil variables were not significant predictors of species abundance within modules but showed a weak yet significant correlation to abundance data for the entire dataset.

## Discussion

### Structure and Drivers of the Plant-Orthopteran Co-occurrence Network

The organization of the plant-orthopteran co-occurrence network in the grasslands of the Jura Mountains was strongly modular. Other examples of modular co-occurrence networks were found among soil microbes (Barberan et al., 2012; Banerjee et al., 2015). However, such organization was not encountered in plants and arbuscular mycorrhizal fungi (AMF) co-occurrence networks (Encinas-Viso et al., 2016). Contrary to this plant-AMF network where random encounters appear to drive species assembly, our results show that the interaction of multiple assembly rules can explain the modular organization of the plant-orthopteran co-occurrence network. For instance, environmental filtering, limiting similarity and neutral processes alternatively explain plant species coexistence within the different modules. To the contrary, within-guild interactions cannot explain the distribution of orthopteran species among modules. It follows that the distribution of orthopterans into modules most likely reflected that of plants as a result of biotic interactions (herbivory, refuges, reproduction sites). This modular structure is likely to shape the response of plant and orthopteran community assembly to future changes, for example, by limiting the negative impact of land-use changes to individual modules leaving other modules un-impacted. Moreover, a limited number of plant or orthopteran species had a disproportionate positive or negative importance for the modular structure of the network. Species with positive importance for the modular structure were generally less connected to other species than species with negative importance. These species are likely to have particular importance for the structure and functioning of grassland ecosystems (Olesen et al., 2007). Our analyses further revealed a complexity in the processes underlying species co-occurrences. For instance, within-guild processes appear to dominate the co-occurrence of plant species while that of orthopterans is best explained by bottom–up processes (i.e., interactions with plants). Below we provide more detailed explanations about how our results support this conclusion for plants and orthopterans, respectively.

In plants, functional trait analyses revealed that different assembly rules operate in the different modules. In module 4, where the functional distances among species were lower than expected by chance, all species had high SLA and low LDMC. This module was composed by seven plant species including *Rumex obtusifolius* and *Aethusa cynapium.* Species in this module are ruderal species able of rapid resource acquisition and are characteristics of grasslands where grazing intensity is high (Cruz et al., 2010). It is likely that species coexistence within this module results from the interplay of the environmental filtering effect of grazing and the competitive exclusion of species unable of rapid resource acquisition. This agrees with Tilman’s Resource Ratio Hypothesis (Tilman, 1982; Miller et al., 2005) where resource acquisition rate determines species coexistence. Species in module 2 were phylogenetically more similar than expected by chance. This result suggests that environmental or biotic filtering forces species to share similar eco-evolutionary features. However, these features were not related to the four investigated traits. To the contrary, plant species co-occurrences were best explained by the limiting similarity process in module 3 where species were functionally less similar than expected by chance. In module 5, the lower phylogenetic relatedness than expected by chance can also be explained by the limiting similarity process. However, this process was not related to the selected functional traits. Species in module 1 show neither significant functional or phylogenetic convergence or divergence nor clear changes in mean functional traits. Such a pattern could result from neutral processes where ecological drift and historical (Fukami, 2015) and spatial contingencies are the main drivers of species assembly. For instance, Lau et al. (2015) showed that phylogenetic founder effect can determine the structure of interaction networks.

In orthopterans, the lack of significant convergence of functional and/or phylogenetic distance among species and the relative low importance of environmental variables suggests that species assembly is not the result of strong environmental filters or competitive interactions. However, changes in functional traits and more specifically in egg deposition strategy suggest that the distribution of orthopteran species into modules results from trophic and other vertical interactions with plants. For instance, species in module 2 tended to lay eggs more frequently in plants as opposed to soil. These species were preferentially encountered in higher elevation grasslands where the microclimate provided by plants could protect eggs from the more constraining environmental conditions (e.g., late freeze). Other types of interactions with plants could also explain the distribution of orthopterans into modules. For instance, plants provide orthopterans with food resources, reproduction and habitat sites and refuge against predators (Pellissier et al., 2011; Ibanez et al., 2013). More generally, co-evolution between plants and orthopterans constitutes another likely explanation for the distribution of orthopterans into modules. For example, it has been shown that the diversification of frugivorous vertebrates was associated with plant fleshy fruit production (Fleming et al., 1987).

### Species Importance for Network Structure

Our analyses further highlighted plant and orthopteran species with particular importance for the structure of the co-occurrence network. Species such as *Metrioptera roeseli* and *Anthriscus sylvestris* had a positive influence on modularity. In other words, the network would become less modular if these species go extinct. These species were found in modules 3 and 4. Species co-occurrence in these two modules was determined by different assembly rules (limiting similarity and filtering, respectively) suggesting that assembly rules are not strong determinant of species role for network structure. However, a common feature of these species is that they share few links with species from other modules. Following the terminology of Olesen et al. (2007) for bipartite interaction networks, these species could be referred to as module hub. Management plans specifically targeting these species are likely to maximize the modularity of the whole system and thereby its capacity to retain the negative impact of perturbations within one or few modules. By contrast, species such as *Leucanthemum vulgare* and *Festuca rubra* had a negative influence on network structure (i.e., the network would become more modular without these species). All of these species belong to module 1. They also tended to have more links than species with positive influence on modularity and were frequently linked to species from different modules. Theoretical studies have shown species interaction networks to be robust to the extinction of poorly connected species but to be sensitive to the loss of highly connected species (Sole and Montoya, 2001; Dunne et al., 2002b). As a result, the extinction of these highly connected species is likely to induce cascading effect within the network.

### Differences with Abundance Data

The rules underlying species assembly differed between co-occurrence and abundance data. Null model analyses suggest that plant abundance was strongly determined by seed mass and LDMC. Species with lower seed mass and LDMC such as *Poa trivialis* reached higher abundances. This result likely reflects a strong competition for space where species producing large propagule numbers and capable of rapid colonization and resource exploitation are dominant. To the contrary, phylogenetic or functional patterns could not explain the abundance of orthopteran species. Here, the two dominant species shared lower elevation sites with *Chorthippus albomarginatus* dominating in predominantly grazed grasslands and *Chorthippus biguttulus* dominating in predominantly mowed grasslands. In higher elevation sites, *Metrioptera roeselii* became dominant most likely because of better adaptations to the more constraining abiotic conditions such as a change of feeding regime during its development or its preference for laying eggs in the vegetation. Finally, RDAs showed that the overall abundance of plants and orthopterans was significantly impacted by agricultural practices and spatial variables. This agrees with previous results obtained for plants in the same system (Mauchamp et al., 2014). Interestingly, the impact of agricultural practices was only significant in modules 2, 4, and 5. It is therefore likely that species in these modules will retain most of the impact of changes in agricultural practices such as intensification leaving other species not or poorly impacted (Krause et al., 2003; Teng and McCann, 2004).

## Conclusion

The combination of co-occurrence network analysis, functional and phylogenetic analyses and multivariate analyses of abundance data constitutes a powerful tool to understand the drivers of species assembly. We highlighted a complexity of processes related to the modular structure of the plant-orthopteran co-occurrence network that differs from those explaining species abundance. We also showed that the modular structure of the network is likely to determine how changes in agricultural practices will influence plant and orthopteran communities. The next step is to understand the importance of this modular structure for the long-term maintenance of grassland ecosystem structure and functions as well as to develop tools to integrate network structure into models to improve their capacity to predict future changes.

## Author Contributions

FG gathered species data. BF gathered functional and phylogenetic data. AM conducted phylogenetic analyses. BF and FG conducted the numerical analyses of the data. All authors contributed to writing.

## Conflict of Interest Statement

The authors declare that the research was conducted in the absence of any commercial or financial relationships that could be construed as a potential conflict of interest.

## Footnotes

1 http://tree.bio.ed.ac.uk/software/seal/
