# Supplementary material for: Multiple Assembly Rules Drive the Co-occurrence of Orthopteran and Plant Species in Grasslands: Combining Network, Functional and Phylogenetic Approaches
Source: Front Plant Sci. 2016 Aug 17;7:1224. doi: 10.3389/fpls.2016.01224 (PMC4987333; doi:10.3389/fpls.2016.01224)
Supplement: Supplementary file 1 [file Table_1.PDF]

Supplementary Material

Multiple assembly rules drive the co-occurrence of orthopteran and plant species in grasslands: combining network, functional and phylogenetic approaches

Bertrand Fournier, Arnaud Mouly, François Gillet

Supplementary Table 1. Orthopteran and plant species retained for the construction of the co-occurrence network. Among the 22 orthopteran and 197 plant species, 9 and 82 were selected because of their positive co-occurrences. Modularity analysis revealed that the network is organized into 5 modules. The table shows the species composition of these modules. The five species having the highest positive impact on network modularity are shown in green; the five ones with the highest negative impact are shown in blue. Nomenclature follows *Fauna Europaea* (de Jong et al., 2014) for orthopteran taxa and *Flora Gallica* (Tison and de Foucault, 2014) for plant taxa.

Taxa	Code	Module membership
**Orthopterans**		
Chorthippus albomarginatus (De Geer, 1773)	Cho.alb	4
Chorthippus biguttulus (Linnaeus, 1758)	Cho.big	1
Chrysochraon dispar (Germar, 1834)	Chr.dis	2
Euthystira brachyptera (Ocskay, 1826)	Eut.bra	2
Mecostethus parapleurus (Hagenbach 1822)	Mec.par	1
Metrioptera roeselii (Hagenbach, 1822)	Met.roe	2
Omocestus viridulus (Linnaeus, 1758)	Omo.vir	2
Stenobothrus lineatus (Panzer, 1796)	Ste.lin	1
Stethophyma grossum (Linnaeus, 1758)	Ste.gro	1
**Plants**		
Achillea millefolium L.	Ach.mil	3
Aethusa cynapium L.	Aet.cyn	4
Agrostis capillaris L.	Agr.cap	3
Ajuga reptans L.	Aju.rep	1
Alchemilla monticola Opiz	Alc.mon	2
Anthoxanthum odoratum L.	Ant.odo	1
Anthriscus sylvestris (L.) Hoffm.	Ant.syl	2
Anthyllis vulneraria L.	Ant.vul	1
Arrhenatherum elatius (L.) P.Beauv. ex J.Presl & C.Presl	Arr.ela	1
Avenula pubescens (Huds.) Dumort.	Ave.pub	1
Bellis perennis L.	Bel.per	1
Bistorta officinalis Delarbre	Pol.bis	2
Briza media L.	Bri.med	1
Bromopsis erecta (Huds.) Fourr.	Bro.ere	1

Bromus hordeaceus L.	Bro.hor	2
Campanula patula L.	Cam.pat	2
Capsella bursa-pastoris (L.) Medik.	Cap.pas	4
Cardamine pratensis L.	Car.pra	1
Carum carvi L.	Car.car.1	2
Centaurea jacea L.	Cen.jac	1
Cerastium fontanum subsp. *vulgare* (Hartm.) Greuter & Burdet	Cer.tri	1
Cirsium arvense (L.) Scop.	Cir.arv	3
Colchicum autumnale L.	Col.aut	1
Convolvulus arvensis L.	Con.arv	4
Crepis biennis L.	Cre.bie	1
Crepis vesicaria subsp. *taraxacifolia* (Thuill.) Thell. ex Schinz & R.Keller	Cre.hae	2
Cynosurus cristatus L.	Cyn.cri	3
Daucus carota L.	Dau.car	3
Elytrigia repens (L.) Desv. ex Nevski	Ely.rep	3
Festuca rubra L.	Fes.rub	1
Galium album Mill.	Gal.alb	1
Heracleum sphondylium L.	Her.sph	2
Holcus lanatus L.	Hol.lan	1
Hypochoeris radicata L.	Hyp.rad	3
Jacobaea vulgaris Gaertn.	Sen.jac	1
Knautia arvensis (L.) Coult.	Kna.arv	1
Koeleria pyramidata (Lam.) P.Beauv.	Koe.pyr	1
Lamium album L.	Lam.alb	4
Lathyrus pratensis L.	Lat.pra	2
Leucanthemum vulgare Lam.	Leu.vul	1
Lolium perenne L.	Lol.per	4
Lotus corniculatus L.	Lot.cor	1
Luzula multiflora (Ehrh.) Lej.	Luz.mul	1
Lychnis flos-cuculi L.	Lyc.cuc	2
Medicago lupulina L.	Med.lup	1
Myosotis arvensis Hill	Myo.arv	1
Phleum pratense L.	Phl.pra	3
Plantago lanceolata L.	Pla.lan	1
Plantago major L.	Pla.maj	3
Plantago media L.	Pla.med	1
Poa pratensis L.	Poa.pra	1
Poa trivialis L.	Poa.tri	5
Polygonum aviculare L.	Pol.avi	4
Potentilla tabernaemontani Asch.	Pot.tab	3
Poterium sanguisorba L.	San.min	1
Primula veris L.	Pri.ver	1
Prunella vulgaris L.	Pru.vul	3
Ranunculus bulbosus L.	Ran.bul	1

Ranunculus acris subsp. *Friesianus* (Jord.) Syme	Ran.fri	5
Ranunculus repens L.	Ran.rep	3
Raphanus raphanistrum L.	Rap.rap	3
Rhinanthus minor L.	Rhi.min	2
Rumex acetosa L.	Rum.ace	2
Rumex obtusifolius L.	Rum.obt	4
Salvia pratensis L.	Sal.pra	1
Sanguisorba officinalis L.	San.off	2
Schedonorus arundinaceus (Schreb.) Dumort.	Fes.aru	3
Schedonorus pratensis (Huds.) P.Beauv.	Fes.pra	3
Sherardia arvensis L.	She.arv	1
Stellaria graminea L.	Ste.gra	2
Stellaria media (L.) Vill.	Ste.med	3
Thymus polytrichus A.Kern. ex Borbás	Thy.pol	1
Tragopogon pratensis subsp. *orientalis* (L.) Celak.	Tra.ori	1
Trifolium pratense L.	Tri.pra	1
Trifolium repens L.	Tri.rep	5
Trisetum flavescens (L.) P.Beauv.	Tri.fla	1
Valerianella locusta (L.) Laterr.	Val.loc	2
Veronica arvensis L.	Ver.arv	2
Veronica chamaedrys L.	Ver.cha	3
Veronica serpyllifolia L.	Ver.ser	3
Vicia angustifolia L.	Vic.nig	1
Vicia cracca L.	Vic.cra	2

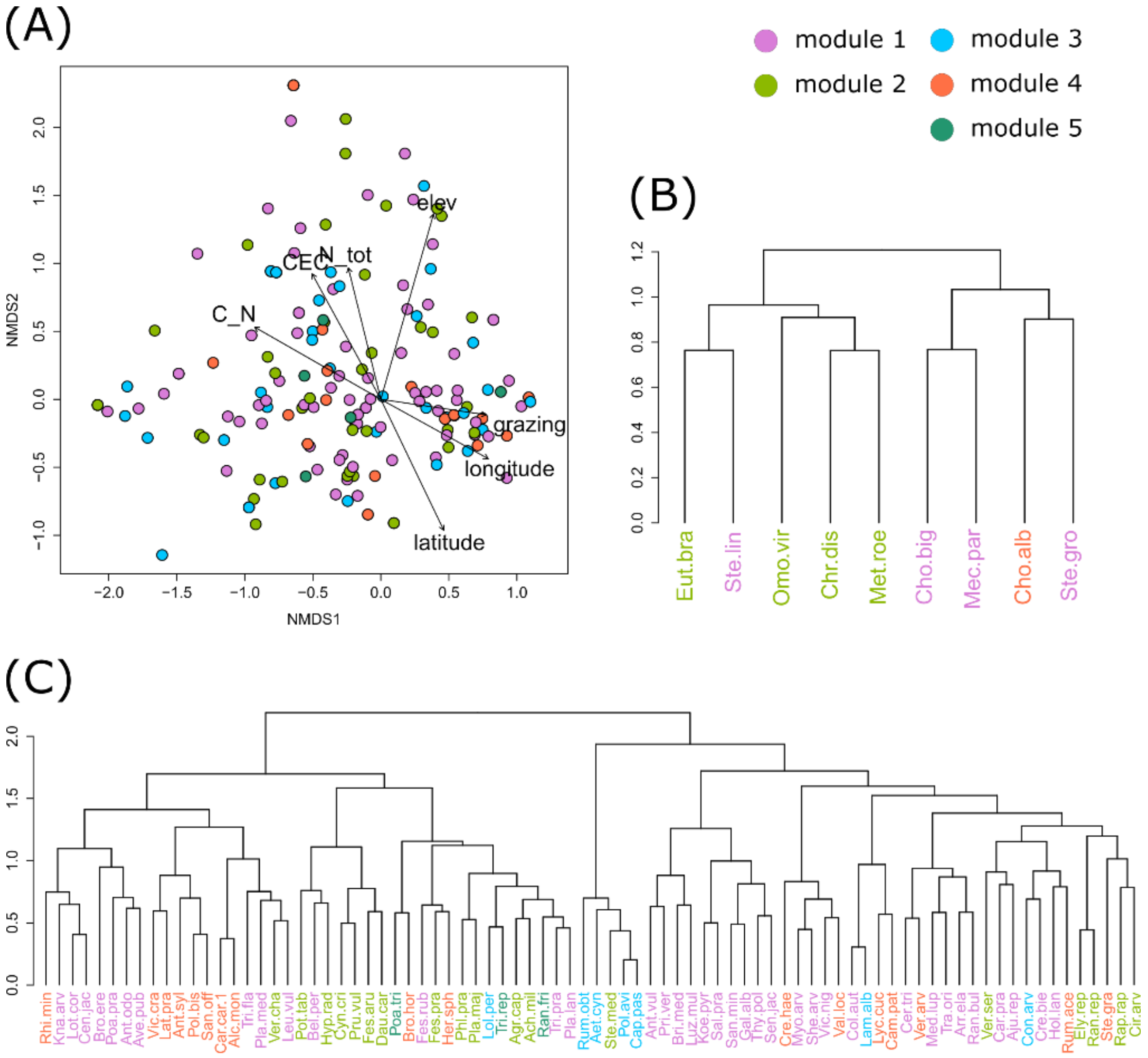

Supplementary Figure 1. Ordination and clustering of plant and orthopteran relative abundance data. **(A)** Nonmetric multidimensional scaling (NMDS) with stable solution from random starts, axis scaling and species scores with environmental variables fitted *a posteriori*. Points correspond to species and point colors show species membership to the five modules (Figure 2). **(B)** Clustering of orthopteran abundance data with species module membership. **(C)** Clustering of plant abundance data with species module membership.

References

de Jong Y., Verbeek M., Michelsen V., Bjørn P., Los W., Steeman F., et al. (2014). Fauna Europaea – all European animal species on the web. *Biodiversity Data Journal* 2: e4034.

Tison J.M. and de Foucault B. (2014). *Flora Gallica – Flore de France*. Mèze: Biotope.
